# Sexual Dimorphism in the Age-Induced Insulin Resistance, Liver Steatosis, and Adipose Tissue Function in Rats

**DOI:** 10.3389/fphys.2017.00445

**Published:** 2017-07-11

**Authors:** Francisco Garcia-Carrizo, Teresa Priego, Nara Szostaczuk, Andreu Palou, Catalina Picó

**Affiliations:** ^1^Laboratory of Molecular Biology, Nutrition and Biotechnology (Nutrigenomics), University of the Balearic Islands Palma de Mallorca, Spain; ^2^CIBER Fisiopatología de la Obesidad y Nutrición (CIBEROBN) Palma de Mallorca, Spain

**Keywords:** sex-differences, white adipose tissue expandability, metabolic disturbances, leptin/adiponectin ratio, MEST

## Abstract

Age-linked metabolic disturbances, such as liver steatosis and insulin resistance, show greater prevalence in men than in women. Thus, our aim was to analyze these sex-related differences in male and female Wistar rats (aged 26 days and 3, 7, and 14 months), and to assess their potential relationship with alterations in the capacity of adipose tissue expansion and the dysregulation of the main adipokines produced by the adipose tissue, leptin and adiponectin. Adiposity-related parameters, blood parameters, the expression of genes related to expandability and inflammation (WAT), lipid metabolism (liver), and leptin and insulin signaling (both tissues) were measured. In females, adiposity index and WAT DNA content gradually increased with age, whereas males peaked at 7 months. A similar sex-dependent pattern was observed for *leptin* expression in WAT, while *Mest* expression levels decreased with age in males but not in females. Females also showed increased expression of the proliferation marker PCNA in the inguinal WAT compared to males. In males, leptin/adiponectin ratio greatly increased from 7 to 14 months in a more acute manner than in females, along with an increase in HOMA-IR index and hepatic triacylglyceride content, while no changes were observed in females. In liver, 14-month-old males displayed decreased mRNA levels of *Insr, Ampk*α*2*, and *Cpt1a* compared with levels at 7 months. Males also showed decreased mRNA levels of *Obrb* (both tissues), and increased expression levels of *Cd68* and *Emr1* (WAT) with age. In conclusion, females are more protected from age-related metabolic disturbances, such as insulin resistance, hepatic lipid deposition, and WAT inflammation compared to males. This may be related to their greater capacity for WAT expansion—reflected by a greater *Mest/leptin* mRNA ratio—and to their ability to maintain adiponectin levels and preserve leptin sensitivity with aging.

## Introduction

Aging is associated with an increased risk of metabolic alterations, such as insulin resistance, hepatic steatosis, and dyslipidemia (Goodpaster et al., 2003; Bertolotti et al., 2014). Notably, such disturbances are more prevalent in men than in women (Krotkiewski et al., 1983; Frias et al., 2001); similarly, in animal studies, less severe diet-induced metabolic disorders have been described in females compared to males, including lower lipid accumulation in liver and greater insulin sensitivity (Priego et al., 2008; Medrikova et al., 2012). However, the cause for sex-dependent differences in the susceptibility to obesity-related metabolic alterations is still unclear.

Fat distribution and function change substantially throughout life, and a possible cause of age-related alterations could be the existence of dysfunctional fat tissue (Tchkonia et al., 2010). In this sense, a low capacity for white adipose tissue (WAT) expansion, i.e., the capacity to form new adipocytes capable of accumulating excess energy and protecting from adipocyte hypertrophy and ectopic lipid accumulation (Hardy et al., 2012), has been linked with greater metabolic disorders in several animal models (Virtue and Vidal-Puig, 2008). The limited capacity of WAT to store energy is accompanied by the accumulation of excess fat in non-adipose tissues, contributing to their dysfunction, and therefore favoring the development of metabolic-syndrome associated disorders (Slawik and Vidal-Puig, 2006). Remarkably, aging is one of the causes of the decrease in adipose tissue capacity to store excess energy, which has been related to the increased prevalence of metabolic alterations in older populations (Rodriguez et al., 2005).

Furthermore, WAT plays a major role as an endocrine tissue, affecting the entire metabolic system, and therefore age-related WAT disturbances may have a great impact on the whole physiology. It releases two key adipokines, leptin and adiponectin, which play central roles in WAT and liver function and are affected by the aging process. On the one hand, leptin production increases with age, which has been related to leptin resistance development (Oliver et al., 2001; Stucchi et al., 2011; Carter et al., 2013). In turn, resistance to leptin action in liver has been proposed as an important determinant of hepatic triacylglyceride (TG) accumulation (Fishman et al., 2007; Stucchi et al., 2011). Notably, under a high-fat diet, female rats have shown to have a greater capacity than males to respond to central and peripheral actions of leptin (Priego et al., 2009a,b). On the other hand, circulating levels of adiponectin are negatively correlated with obesity and the presence of diabetes (Hotta et al., 2000), and have been shown to improve insulin sensitivity and glycemic control (Yamauchi and Kadowaki, 2013), increase expansion of adipose tissue under chronic high-fat diet feeding (Asterholm and Scherer, 2010), and prevent the development of fatty liver disease (Awazawa et al., 2009; Asterholm and Scherer, 2010). Thus, the potential sex-dependent differences in adiponectin profile could also be of interest.

Considering the abovementioned, we aimed to characterize the sex differences in age-related metabolic disturbances in rats, particularly liver steatosis, insulin resistance, and in the dysregulation of leptin and adiponectin, together with the assessment of adipose tissue expansion capacity, in order to get some insight into the potential mechanism involved.

## Materials and methods

### Animals and experimental design

The study was performed in male and female Wistar rats (Charles River Laboratories, Barcelona, Spain). All animals were housed under controlled temperature (22°C) and a 12 h light–dark cycle (light on from 08:00 to 20:00), and had unlimited access to tap water and standard chow diet (3.2 kcal/g, with 8% calories from fat; Panlab, Barcelona, Spain). The animal protocol followed in this study was reviewed and approved by the Bioethical Committee of the University of the Balearic Islands (Resolution Number 8453. June 2010), following its guidelines for the use and care of laboratory animals. All efforts were made to minimize suffering.

Animals of both sexes were killed at the age of 26 days (*n* = 12), 3 (*n* = 10), 7 (*n* = 10), and 14 (*n* = 10) months by decapitation under fed conditions. Liver and WAT depots (gonadal, gWAT; retroperitoneal, rWAT; mesenteric, mWAT; and inguinal, iWAT) were rapidly removed. All samples were weighed and immediately frozen in liquid nitrogen and stored at –70°C. The iWAT (subcutaneous depot) and the rWAT (internal depot) were chosen to measure mRNA expression of selected genes at the different ages studied, because of the known differences in gene expression patterns and morphological features between subcutaneous and internal depots (Palou et al., 2009). Morphometric and immunohistochemical analyses were performed in the four studied depots at the age of 14 months.

### Measurement of circulating parameters

One week prior to sacrifice, blood samples were collected from the saphenous vein under 14 h fasting conditions, without anesthesia and during the first 2 h after the beginning of light phase. Samples were centrifuged (700 g, 10 min) in order to obtain serum, and stored at –20°C until analysis. Blood glucose concentration was measured by Accu-Chek Glucometer (Roche Diagnostics, Barcelona, Spain). Serum insulin concentration was determined using a rat insulin enzyme-linked immunosorbent assay (ELISA) kit (Mercodia AB, Uppsala, Sweden), and leptin and adiponectin concentrations were measured using the ELISA kit QuantikineTM Mouse Leptin Inmunoassay and ELISA kit QuantikineTM Rat Total Adiponectin/Acrp30, respectively (R&D Systems, Minneapolis, MN, USA). Plasma TG levels were measured using the Serum Triglyceride Determination Kit (Sigma Aldrich).

The homeostatic model assessment for insulin resistance (HOMA-IR) was used to assess insulin resistance, using the formula HOMA-IR = fasting glucose (mmol/liter) × fasting insulin (mU/liter)/22.5 (Matthews et al., 1985).

### Hepatic triacylglycerol determination

Liver samples (100–200 mg) were homogenized in phosphate saline buffer (PBS; 1/3, w/v) and centrifuged (500 g, 10 min). The supernatant was then used for hepatic TG quantification using the Serum Triglyceride Determination Kit (Sigma Aldrich).

### Quantification of DNA levels

Fifty milligrams of different WAT depots (iWAT, gWAT, mWAT, and rWAT) were homogenized in PBS (1:9, w:v) and centrifuged (500 g, 10 min). The supernatant was collected and used for DNA quantification following a fluorometric method that employs 3,5 diaminobenzoic acid (Kissane and Robins, 1958).

### Morphometric and immunohistochemical analyses

Morphometric and immunohistochemical analyses were performed in the four WAT depots from 14-month-old rats. Fragments of tissues were fixed in 4% paraformaldehyde in 0.1M phosphate buffer pH 7.3 overnight at 4°C, and then washed in the same buffer. For paraffin embedding, samples were dehydrated in ethanol, cleared in xylene, and embedded in paraffin blocks at 60°C.

For immunohistochemistry analysis, 5 μm sections were immunostained by means of the avidin-biotin technique using a commercial anti-MAC2 antibody (1:350 in PBS, Cederlane, Hornby, Ontario, Canada), counterstained with hematoxylin and mounted in Eukitt (Kindler, Freiburg, Germany). Images were acquired with a Zeiss Axioskop 2 microscope equipped with AxioCam ICc3 digital camera and AxioVision 40V 4.6.3.0 Software (Carl Zeiss, S.A., Barcelona, Spain). The software was also used to measure the diameter of 100 adipocytes (from one random field) in two non-consecutive hematoxylin/eosin stained sections. Image analysis from all groups was examined in a blind fashion.

### RNA extraction

Total RNA was extracted from liver and two depots of WAT (iWAT and rWAT) by using the EZNA® TOTAL RNA kit I (Omega Bio-Tek Inc., Norcross, GA, USA) according to the manufacturer's instructions. Isolated RNA was quantified using the NanoDrop ND-1000 spectrophotometer (NadroDrop Technologies Inc., Wilmington, Delaware, USA), and its integrity confirmed using 1% agarose gel electrophoresis.

### Reverse transcription quantitative polymerase chain reaction (RT-qPCR) analysis

0.25 μg of total RNA (in a final volume of 5 μl) was denatured (65°C, 10 min) and then reverse transcribed to cDNA using MuLV reverse transcriptase (Applied Biosystems, Madrid, Spain) at 20°C for 15 min, 42°C for 30 min, with a final step of 5 min at 95°C in a Thermal Cycler (Applied Biosystems). Each qPCR was performed from diluted cDNA template, forward and reverse primers (1 μM each), and Power SYBER Green PCR Master Mix (Applied Biosystems, CA, USA). Primers were obtained from Sigma Genosys (Sigma-Aldrich Quimica SA, Madrid, Spain; Table 1). Real time PCR was performed using the StepOnePlusTM Real-Time PCR Systems (Applied Biosystems) with the following profile: 10 min at 95°C, followed by a total of 40 two-temperature cycles (15 s, 95°C and 1 min, 60°C). In order to verify the purity of the products, a melting curve was produced after each run according to the manufacturer's instructions. The threshold cycle (Ct) was calculated by the instrument's software (StepOne Software v2.2.2), and the relative expression of each mRNA was calculated as a percentage of 26-day-old male rats, using the 2^−ΔΔCt^ method (Pfaffl, 2001). Low-density lipoprotein receptor related protein 10 (*Lrp10*) (in liver) and guanosine diphosphate dissociation inhibitor 1 (*Gdi1*) (in both adipose tissue depots) were used as reference genes. They were previously validated to ensure they had stable expression levels at different ages and in both sexes in the referred tissues.

**Table 1:** Nucleotide sequences of primers used for PCR amplification.

**Gene**	**Forward Primer (5′–3′)**	**Reverse Primer (5′–3′)**	**Amplicon size (bp)**
*Adipor1*	AGCACCGGCAGACAAGAG	GGTGGGTACAACACCACTCA	68
*Ampkα2*	CCAAGTGATCAGCACTCCAA	CAACACGTTCTCTGGCTTCA	199
*Cd68*	AATGTGTCCTTCCCACAAGC	GGCAGCAAGAGAGATTGGTC	233
*Cpt1a*	GCAAACTGGACCGAGAAGAG	CCTTGAAGAAGCGACCTTTG	180
*Gdi1*	CCGCACAAGGCAAATACATC	GACTCTCTGAACCGTCATCAA	210
*Emr1*	CTCTTCTGGGGCTTCAGTGG	CTCTTCTGGGGCTTCAGTGG	204
*Insr*	GTCCGGCGTTCATCAGAG	CTCCTGGGATTCATGCTGTT	242
*Leptin*	TTCACACACGCAGTCGGTAT	AGGTCTCGCAGGTTCTCCAG	186
*Lrp10*	CGGATGGAGGCTGAGATTG	AGCACAGAGTTGTCATTGGG	122
*Mest*	CTCAGCTCTCCCCTGCTCT	GCAATCACTCGATGGAACCT	202
*Obrb*	AGCCAAACAAAAGCACCATT	TCCTGAGCCATCCAGTCTCT	174
*Pcna*	TATTGGAGATGCTGTGGTGA	AGTGGAGTGGCTTTTGTGAA	204
*Rn18s*	CGCGGTTCTATTTTGTTGGT	AGTCGGCATCGTTTATGGTC	219
*Srebf1*	AGCCATGGATTGCACATTTG	GGTACATCTTTACAGCAGTG	260

### Statistical analyses

All data are expressed as mean ± standard error of the mean (SEM). Statistical analysis between groups were made by a two-way ANOVA (with sex and age as factors) followed by a one-way ANOVA, and least significant difference (LSD) *post-hoc* test was used when differences were statistically significant (*P* < 0.05). Analysis of log-transformed data was performed when variances were heterogeneous. Single differences between male and female rats at the same age were assessed by Student's *t*-test. Distribution of adipocytes by size (diameter) was analyzed by the quantitative distribution method. The comparison of distributions between males and females of 14-months of age was determined by Wilcoxon rank sum test. All analyses were performed using SPSS 21 for Windows.

## Results

### Body weight, adiposity index, blood parameters, and hepatic triacylglyceride content

**Figure 1:**
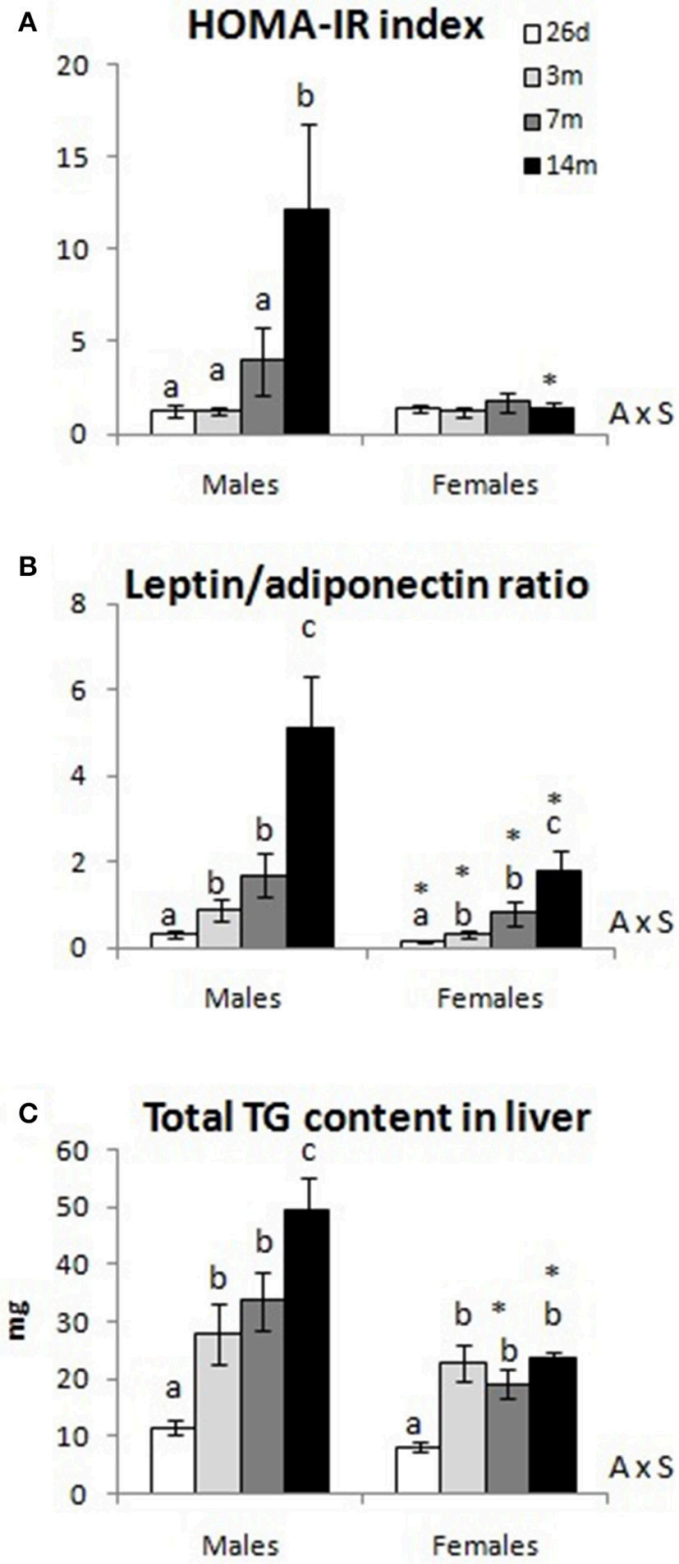
HOMA-IR index **(A)**, circulating leptin/adiponectin ratio **(B)**, and total triacylglyceride (TG) content in liver **(C)** of male and female animals at 26 days, 3, 7, and 14 months. Data represent mean ± SEM. Statistics: AxS interaction between age and sex by two-way ANOVA, a ≠ b ≠ c by LSD *post-hoc* analysis. ^*^different from males at the same age by Student's *t*-test.

As shown in Table 2, body weight in both males and females gradually increased with age. Similarly, adiposity index increased with age in both sexes, but in males this reached a stable level at 7 months (interaction between sex and age, *P* < 0.05 by two-way ANOVA). Fasting glucose levels were greater in 26-day-old animals, both males and females, compared to those at 3 and 7 months; interestingly, an increase of fasting glucose levels in rats at early ages compared to 6-month-old ones has been previously described (Palou et al., 2012). At the age of 14 months, males, but not females, showed increased fasting glucose levels compared to 3- and 7-month-old animals, reaching levels not different from those at 26 days (interaction between sex and age, *P* < 0.05 by two-way ANOVA). Regarding fasting insulin, male animals showed a gradual increase in their levels with age, whereas females showed no differences with age (interaction between sex and age, *P* < 0.05 by two-way ANOVA). Sex-differences were also observed regarding the assessment of insulin resistance (Figure 1A): whereas HOMA-IR index increased in male animals with age, particularly from 7 to 14 months, no changes were observed in females (interaction between sex and age, *P* < 0.05 by two-way ANOVA). In addition, 14-month-old female animals displayed a lower HOMA-IR value than males at the same age.

**Table 2:** Body weight, adiposity index, and blood parameters in male and female animals analyzed at 26 days, 3, 7, and 14 months.

	**Males**				**Females**				
	**26 days**	**3 months**	**7 months**	**14 months**	**26 days**	**3 months**	**7 months**	**14 months**	**Two-way ANOVA**
Weight (g)	75.0^a^ ± 4.6	356^b^ ± 11	475^c^ ± 16	532^d^ ± 20	70.0^a^ ± 2.1	221^b^^*^ ± 5	259^c^^*^ ± 5	302^d^^*^ ± 6	AxS
Adiposity index (g/100g)	2.36^a^ ± 0.22	4.41^b^ ± 0.46	8.48^c^ ± 0.95	9.40^c^ ± 0.80	2.20^a^ ± 0.208	3.01^a^^*^ ± 0.24	6.60^b^ ± 0.38	9.21^c^ ± 0.85	AxS
Fasting glucose (mg/dL)	105^a^ ± 5	64^b^ ± 2	75^b^ ± 7	115^a^ ± 16	104^a^ ± 7	75^b^ ± 7	77^b^ ± 5	85^b^ ± 8	AxS
Fasting insulin (μg/L)	0.21^a^ ± 0.05	0.34^a^ ± 0.05	0.84^ab^ ± 0.37	1.64^b^ ± 0.38	0.22 ± 0.03	0.26 ± 0.05	0.39 ± 0.11	0.27^*^ ± 0.04	AxS
Leptin (ng/L)	888^a^ ± 230	1773^ab^ ± 394	3249^b^ ± 502	5363^c^ ± 1021	242^a^^*^ ± 52	1093^b^ ± 159	1231^b^^*^ ± 304	2798^c^^*^ ± 539	A, S
Adiponectin (μg/L)	2629^a^ ± 259	1762^b^ ± 168	1678^b^ ± 165	931^c^ ± 165	2008^ab^^*^ ± 98	3042^a^^*^ ± 457	1644^ab^ ± 373	1413^b^^*^ ± 184	AxS
TG (mg/ml)	1.10^a^ ± 0.14	1.55^ab^ ± 0.15	1.95^bc^ ± 0.13	2.27^c^ ± 0.28	1.48 ± 0.21	1.10 ± 0.20	1.82 ± 0.27	1.65^*^ ± 0.19	A, S

*Data represent mean ± SEM. Statistics: AxS, interaction between age and sex; A, effect of age; S, effect of sex by two-way ANOVA; a ≠ b ≠ c by LSD post-hoc analysis*.

* *different from males at the same age by Student's t-test. TG, triacylglycerides*.

Leptin levels showed a gradual increase with age in both male and female animals, although levels in females were lower than those of males at the different ages (except at 3 months, when differences did not reach statistical significance; Table 2). Regarding adiponectin levels, male animals showed a gradual decrease with age, and, at the age of 14 months, levels were 3-fold lower than those at 26 days (Table 2). In females, adiponectin levels peaked at 3 months, and decreased moderately thereafter (differences were only significant when comparing levels at 3 and 14 months). At 14 months, female animals showed higher adiponectin levels than males at the same age (interaction between sex and age, *P* < 0.05 by two-way ANOVA). The leptin/adiponectin ratio increased gradually with age in both males and females, but the increase was more marked in males (Figure 1B). Moreover, the levels found in males were around three times higher than those in age-matched females (interaction between sex and age, *P* < 0.05 by two-way ANOVA).

Plasma TG levels increased gradually with age, particularly in male animals, with levels at 14 months greater than those of females at the same age (Table 2). In turn, total TG content in liver also increased gradually with age in males, whereas females exhibited a significant increase at 3 months, but levels remained unchanged afterwards (interaction between sex and age, *P* < 0.05 by two-way ANOVA, Figure 1C). The levels of TG reached by males at 7 and 14 months of age were significantly greater than the ones of females at the same ages.

### WAT weight and DNA content

**Figure 2:**
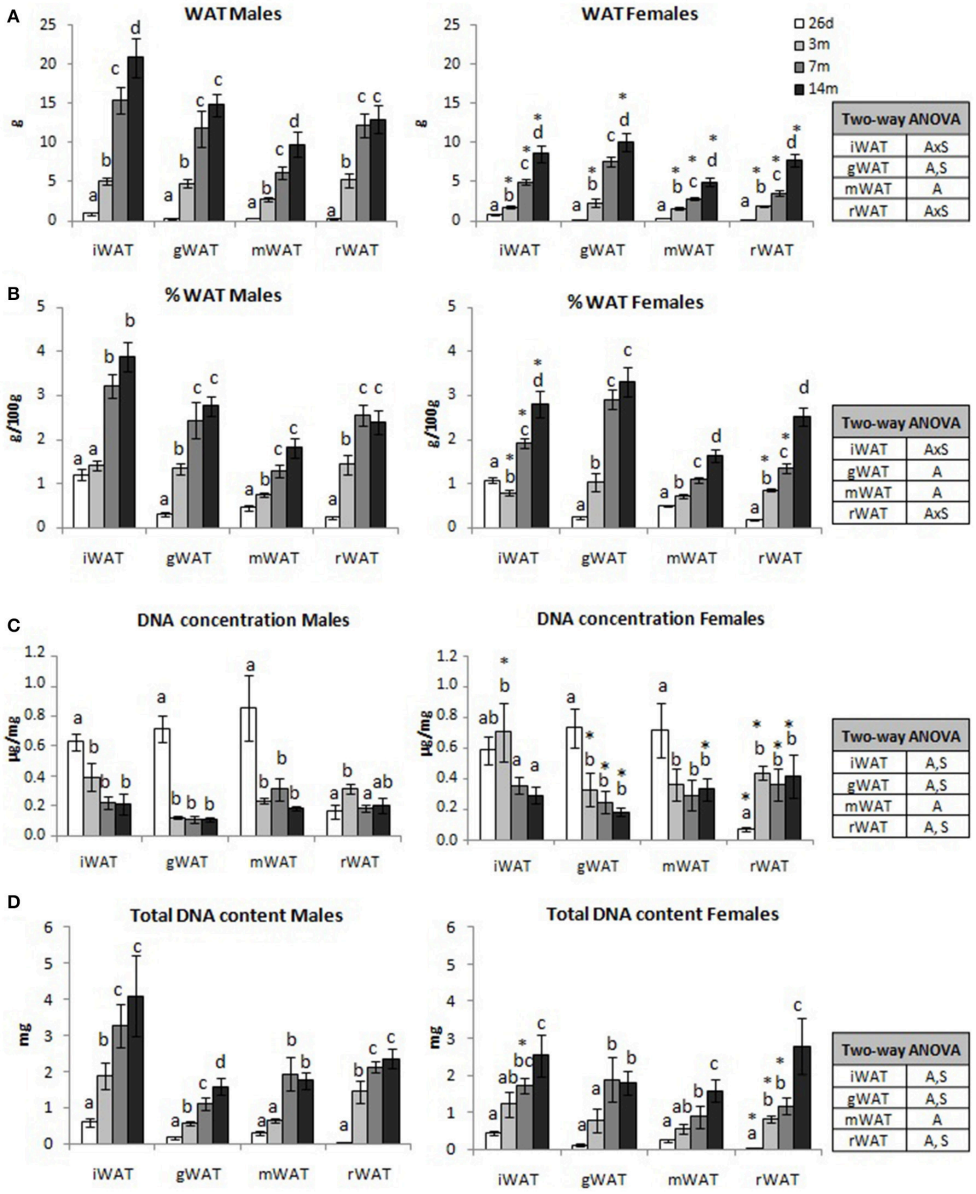
Weight and DNA content of adipose tissue depots. Absolute **(A)** and relative weight **(B)**, DNA concentration **(C)**, and total DNA content **(D)** of inguinal, gonadal, mesenteric, and retroperitoneal white adipose tissue (iWAT, gWAT, mWAT, and rWAT, respectively) of male and female animals at different ages (26 days, 3, 7, and 14 months). Data represent mean ± SEM. Statistics: AxS, interaction between age and sex; A, effect of age; S, effect of sex by two-way ANOVA; a ≠ b ≠ c ≠ d by LSD *post-hoc* analysis. ^*^different from males at the same age by Student's *t*-test.

Absolute and relative WAT weight of the different depots studied (iWAT, gWAT, mWAT, and rWAT) increased with age in both sexes (Figures 2A,B, respectively). However, in male animals the relative weight of each depot reached stable levels at 7 months. This pattern was also observed with the absolute weight of gonadal and retroperitoneal depots. In females, the increase with age was gradual, and absolute and relative weights of the different depots (with the exception of the relative weight of gWAT) were greater at 14 months than at 7 months. Notably, in females, the relative weight of the iWAT depot at 26 days of age was higher than at 3 months. An interaction between sex and age, *P* < 0.05 by two-way was found for the iWAT and rWAT regarding absolute and relative weights.

A similar pattern was observed for total DNA content in WAT. In males (Figure 2D), DNA content of the different depots increased until the age of 7 months and remained constant thereafter, with the exception of gWAT, whose DNA content continued increasing with age. In turn, females showed a gradual increase in DNA content with age in all depots analyzed except in gWAT, whose DNA content remained stable from 7 months onwards. However, DNA concentration (expressed in μg/mg of tissue) decreased with age in male and female animals (with the exception of rWAT), probably reflecting an increase in adipocyte size (Figure 2C). Nevertheless, female animals compared with males showed higher DNA concentration at 3 months (in gWAT and rWAT), 7 months (in all depots with the exception of mWAT), and at 14 months (in all depots with the exception of iWAT).

### WAT morphology and immunohistochemistry

**Figure 3:**
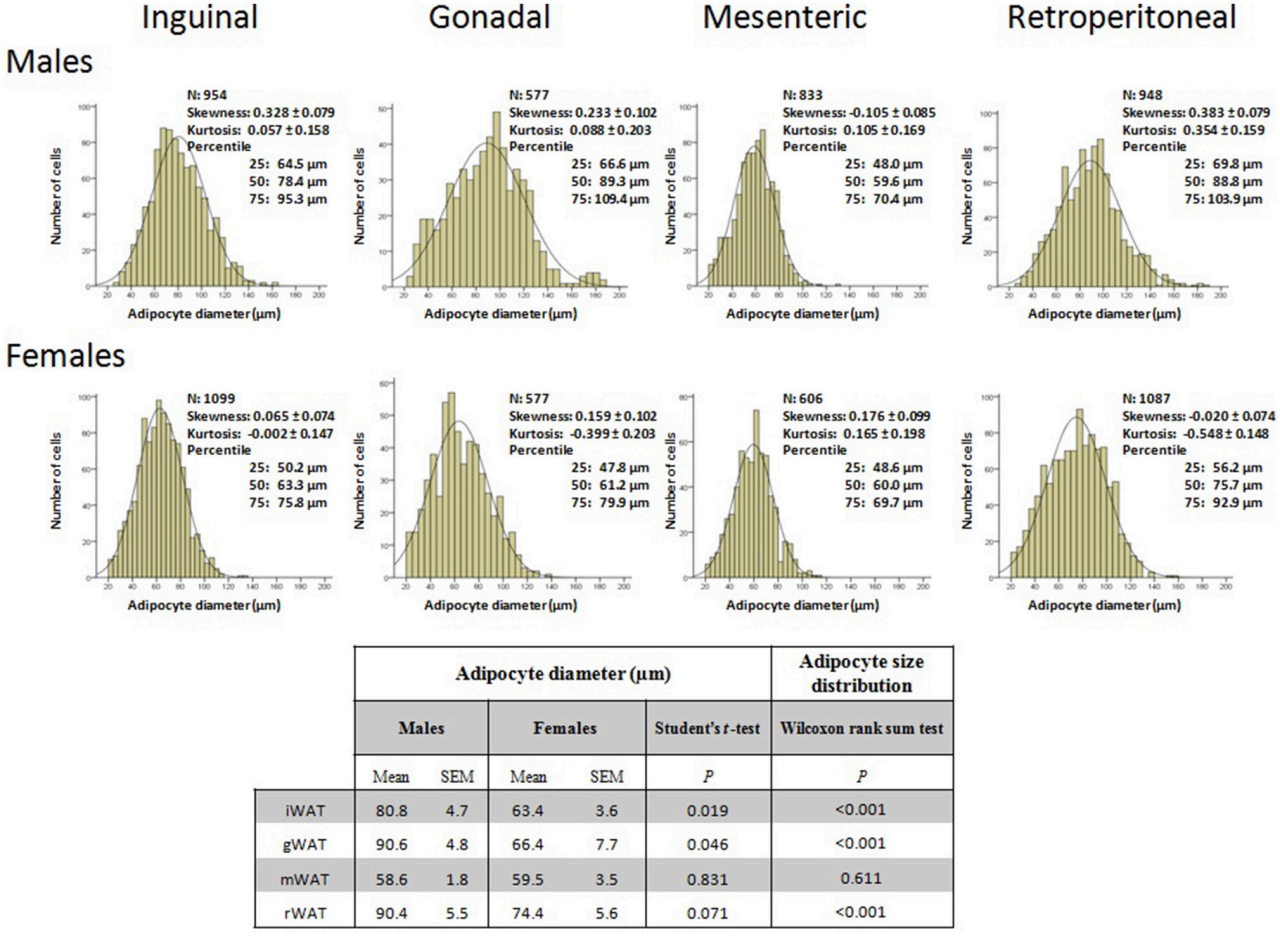
Analysis of the adipocyte size distribution of inguinal, gonadal, mesenteric, and retroperitoneal white adipose tissue (iWAT, gWAT, mWAT, and rWAT, respectively) of 14-month-old animals. Distributions of adipocytes size were obtained from individual data of cell sizes and analyzed by the Quantitative Distribution Method. The number of cells analyzed (n), and features of the distribution (skewness, kurtosis, percentiles) are shown. The diameter of individual adipocytes was measured using a quantitative morphometric method at 20 magnification with the assistance of Axio Vision software. The mean ± SEM of adipocyte diameter, *P*-values of Student's *t*-test and *P*-values of Wilcoxon rank sum test for comparison between males and females (of the mean and of the distributions, respectively) are shown in the table.

Morphometric analysis of samples from 14-month-old rats revealed that, on average, males displayed larger adipocytes than females in iWAT and gWAT depots (Figure 3). A similar tendency was observed in the rWAT depot, but differences did not reach statistical significance (*p* = 0.07, Student's *t*-test). No differences between males and females were observed for the mWAT depot. Differences between males and females became more apparent when quantifying adipose cell size distribution. Accordingly, a shift in adipocyte distribution toward a smaller size was observed in the iWAT, gWAT, and rWAT depots of females in comparison to males (Figure 3). The Wilcoxon rank sum test demonstrated that the difference in distributions of cell size between sexes in the mentioned depots was statistically significant (*P* < 0.0001).

**Figure 4:**
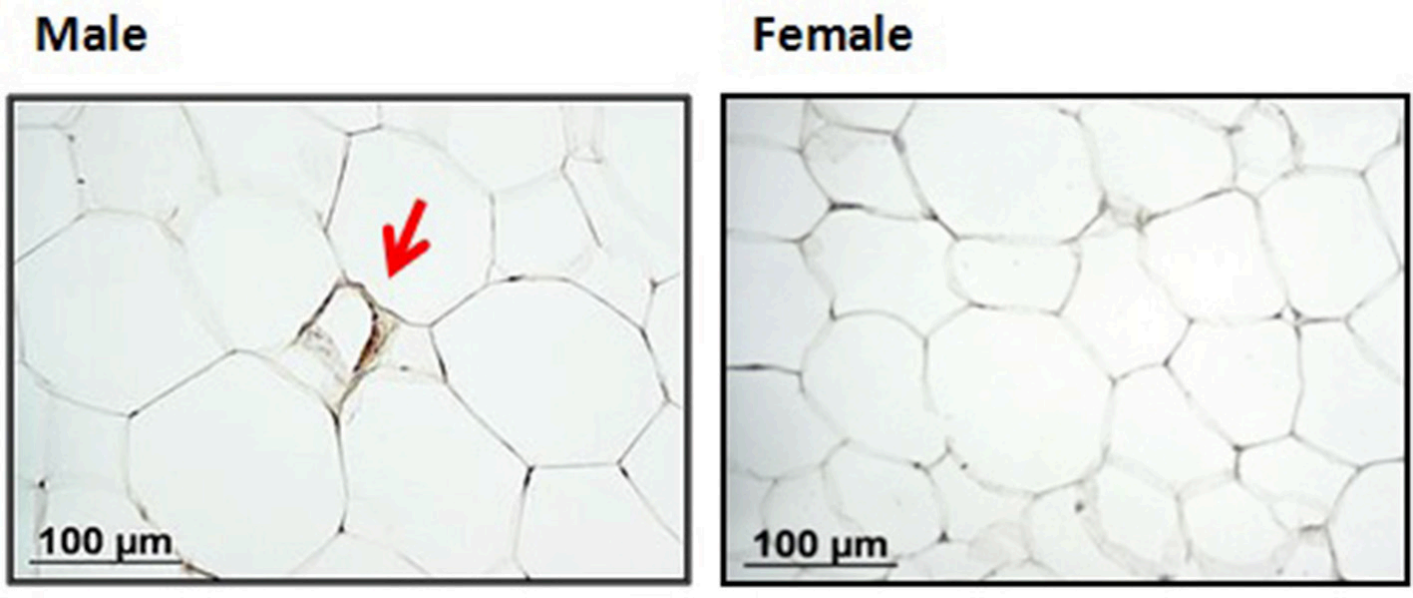
Representative adipose tissue sections (inguinal depot) of 14-month-old male (left) and female (right) animals immunostained for MAC-2 to detect macrophage infiltration. MAC-2-positive macrophages aggregated in crown-like structures found in male animals are indicated by arrows. Sections were counterstained with hematoxilyn-eosin stain. Scale bar 100 μm.

Concerning macrophage infiltration, immunohistochemistry analysis of 14-month-old animals showed the presence of scarce MAC-2-positive macrophages aggregated in crown-like structures (CLS) surrounding individual adipocytes in the iWAT and rWAT depots of male rats. More specifically, one MAC-2-positive CLS was found in the inguinal depot in one of the five animals studied (in an average area of 8.6 × 10^6^ μm^2^) and in two of the five animals analyzed in the case of the retroperitoneal depot (in an average area of 12.8 × 10^6^ μm^2^). However, MAC-2 staining was negative in both depots of female rats, in an average area of 13.2 × 10^6^ μm^2^ and 21.1 × 10^6^ μm^2^ in iWAT and rWAT, respectively (see Figure 4 for a representative image of iWAT). In the gWAT and mWAT depots, only scarce isolated MAC-2-positive macrophages, but not CLS, were found in few animals, with no apparent differences between males and females.

### Gene expression in inguinal and retroperitoneal WAT

**Figure 5:**
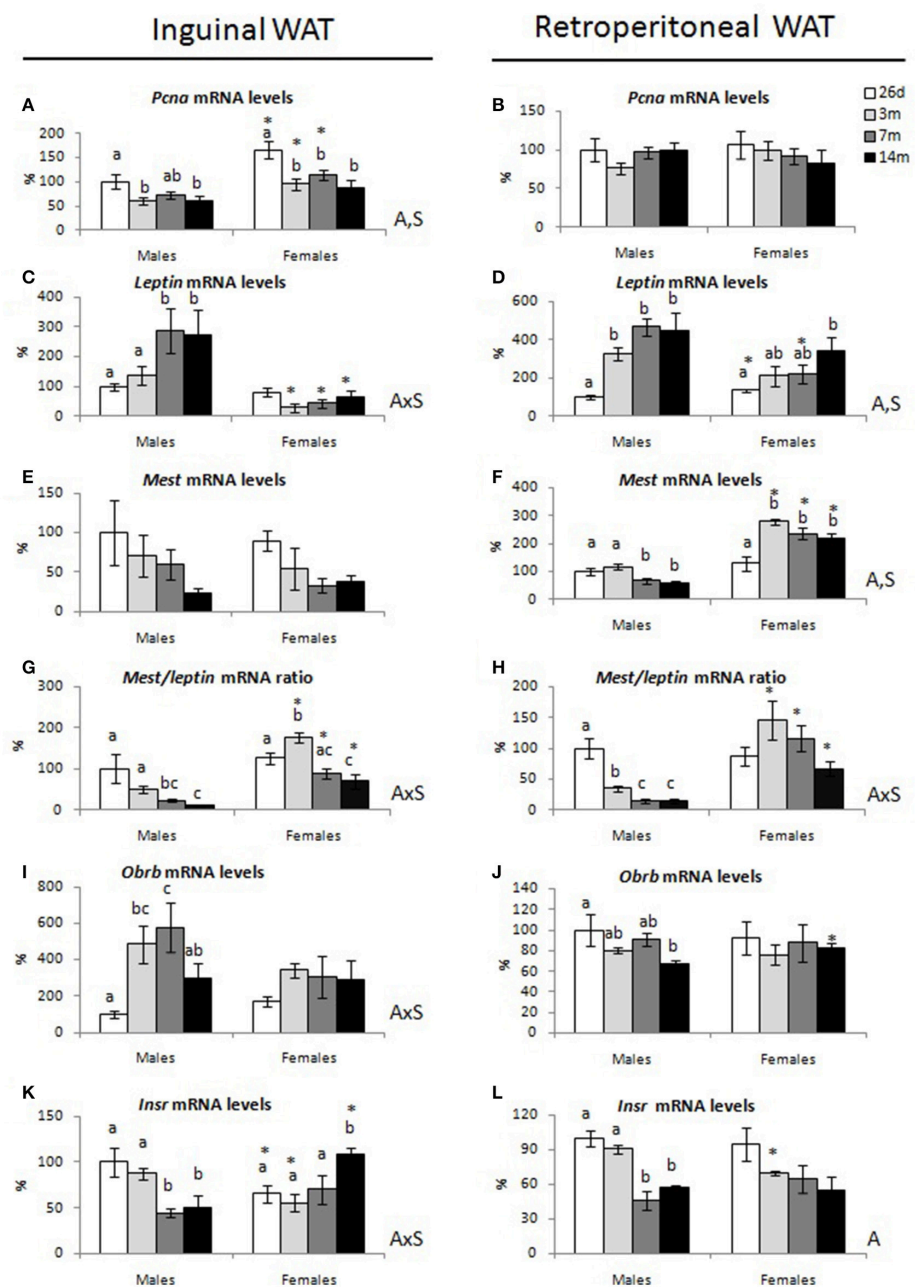
Gene expression levels in inguinal and retroperitoneal WAT of male and female animals at 26 days, 3, 7, and 14 months. **(A,B)** proliferating cell nuclear antigen (Pcna), **(C,D)** leptin, **(E,F)** mesoderm-specific transcript (Mest), **(G,H)** Mest/leptin ratio, **(I,J)** leptin receptor (Obrb), and **(K,L)** insulin receptor (Insr). mRNA levels and the mRNA ratio are expressed as a percentage of the mean values of 26-day-old males. Data represent mean ± SEM. Statistics: AxS, interaction between age and sex; A, effect of age; S, effect of sex by two-way ANOVA; a ≠ b ≠ c by LSD *post-hoc* analysis. ^*^different from males at the same age by Student's *t*-test.

The expression levels of selected genes related to cell proliferation—*proliferating cell nuclear antigen (Pcna)—*adiposity and adipose tissue expansion—*leptin* and *mesoderm specific transcript* (*Mest*)—and with leptin and insulin signaling—*leptin receptor* (*Obrb) and insulin receptor (Insr*)—were studied in inguinal and retroperitoneal WAT depots of male and female animals at different ages (Figure 5).

Regarding the cell proliferation marker *Pcna*, its expression levels in the inguinal depot decreased at the age of 3 months in both males and females and remained constant afterwards. However, the expression levels of this marker were greater in females compared to males at all the studied ages except at 14-months old (Figure 5A). No differences concerning the expression level of *Pcna* were observed in the retroperitoneal depot (Figure 5B).

In males, *leptin* mRNA expression levels in the inguinal depot increased from 3 to 7 months of age and levels were maintained thereafter (Figure 5C). Similarly, in the rWAT depot (Figure 5D), a significant increase was observed from 26 days to 3 months of age, and levels remained unchanged afterwards. In females, *leptin* mRNA expression levels did not change significantly with age in the iWAT depot, while in the rWAT depot, the expression levels increased gradually with age. When comparing females to males, the expression levels of *leptin* in the iWAT depot were significantly lower in females at 3, 7, and 14 months of age (interaction between sex and age, *P* < 0.05 by two-way ANOVA). Nevertheless, in the rWAT depot, females showed higher expression levels of *leptin* at 26 days but lower levels at 7 months of age.

*Mest* mRNA levels in the iWAT depot showed no significant changes with age, although there was a downward trend in both sexes (Figure 5E). In the rWAT depot, male animals exhibited lower *Mest* mRNA levels at 7 and 14 months old compared to those at younger ages (Figure 5F), while females showed increased *Mest* expression levels at 3, 7, and 14 months of age compared to those at 26 days. Notably, expression levels of *Mest* in the rWAT depot of adult females were higher compared to those of males at the same ages.

Considering that *leptin* expression in the adipose tissue is known to be related to adiposity and adipocyte size (Oliver et al., 2001), while *Mest* expression is considered a predictive marker of adipose tissue expansion (Nikonova et al., 2008), the *Mest/Leptin* mRNA ratio was calculated as an indicator of the storage capacity of the adipose tissue (Figures 5G,H). Males displayed a significant decrease in the *Mest/leptin* mRNA ratio with age in both WAT depots analyzed. In females, this ratio peaked at 3 months in iWAT, and levels decreased thereafter. A similar tendency, but not significant, was observed in rWAT. Notably, females showed greater *Mest/leptin* mRNA ratio than males from 3 months on in both WAT depots studied. An interaction between sex and age, *P* < 0.05 by two-way ANOVA, was found regarding *Mest/leptin* mRNA ratio in both iWAT and rWAT.

Expression levels of *leptin receptor* (*Obrb) and insulin receptor (Insr*) were also studied in inguinal and retroperitoneal WAT depots of male and female rats at different ages (Figures 5I,J). In male animals, *Obrb* mRNA levels in the iWAT depot increased with age, peaked at 7 months and decreased thereafter, whereas in the rWAT depot, levels decreased with age, and were significantly different at 14 months compared to those at 26 days. No age-related changes were observed in females concerning *Obrb* expression levels, either in iWAT or rWAT. An interaction between sex and age was found in the iWAT, *P* < 0.05 by two-way ANOVA. Concerning *Insr* (Figures 5K,L), males showed similar expression levels of this gene in both depots at 26 days and 3 months, but levels decreased at 7 months and remained low thereafter. However, in females, *Insr* mRNA levels in the iWAT depot increased at 14 months of age compared with younger ages. They showed at this age higher *Insr* mRNA levels compared with those of males at the same age, but lower levels at 26 days (interaction between sex and age, *P* < 0.05 by two-way ANOVA). In the rWAT, both males and females showed decreased expression levels of *Insr* mRNA expression with age, but changes were more marked and significant only in males.

**Figure 6:**
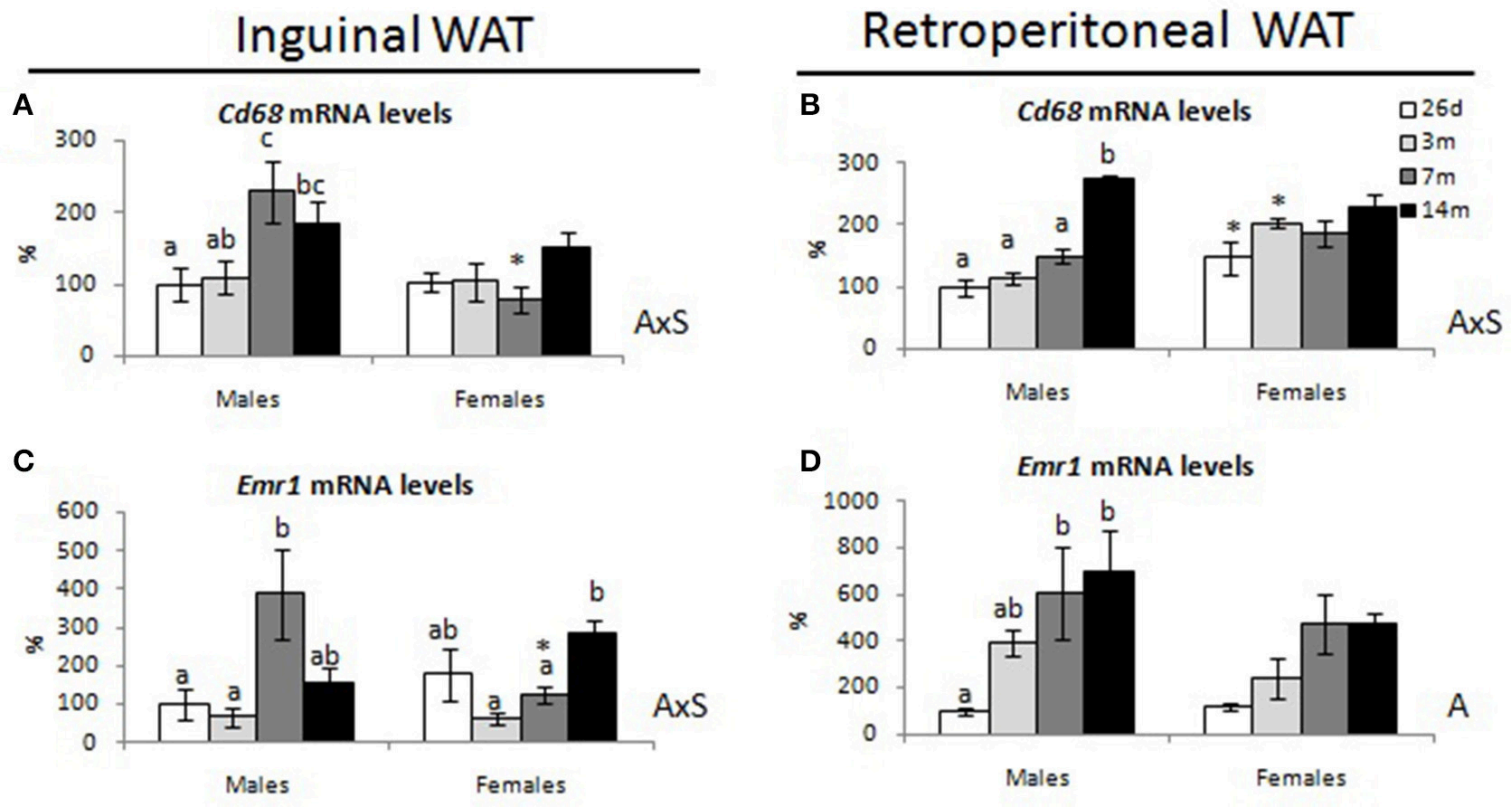
Gene expression levels of macrophage markers in inguinal and retroperitoneal WAT depots of male and female animals at 26 days, 3, 7, and 14 months. **(A,B)** cluster of differentiation 68 (Cd68) and **(C,D)** epidermal growth factor module-containing mucin-like receptor 1 (Emr1) mRNA levels are expressed as a percentage of the mean values of 26-day-old males. Data represent mean ± SEM. Statistics: AxS, interaction between age and sex; A, effect of age by two-way ANOVA; a ≠ b ≠ c by LSD *post-hoc* analysis. ^*^different from males at the same age by Student's *t*-test.

The expression of macrophage-specific markers—*cluster of differentiation 68* (*Cd68*), and *epidermal growth factor module-containing mucin-like receptor 1 (Emr1)*—were also analyzed in the inguinal and retroperitoneal WAT depots. Male animals exhibited greater expression levels of these genes at 7 months in the iWAT depot (Figures 6A,B, respectively) and at 7 and/or 14 months in the rWAT depot (Figures 6C,D, respectively) in comparison with expression levels at younger ages. However, female animals showed no significant changes with age concerning the expression levels of these genes, with the exception of *Emr1* in the iWAT depot, which showed greater expression levels at 14 months than at 3 and 7 months. An interaction between sex and age was found for *Cd68* in both iWAT and rWAT, and for *Emr1* in the iWAT, *P* < 0.05 by two-way ANOVA.

### Gene expression in liver

**Figure 7:**
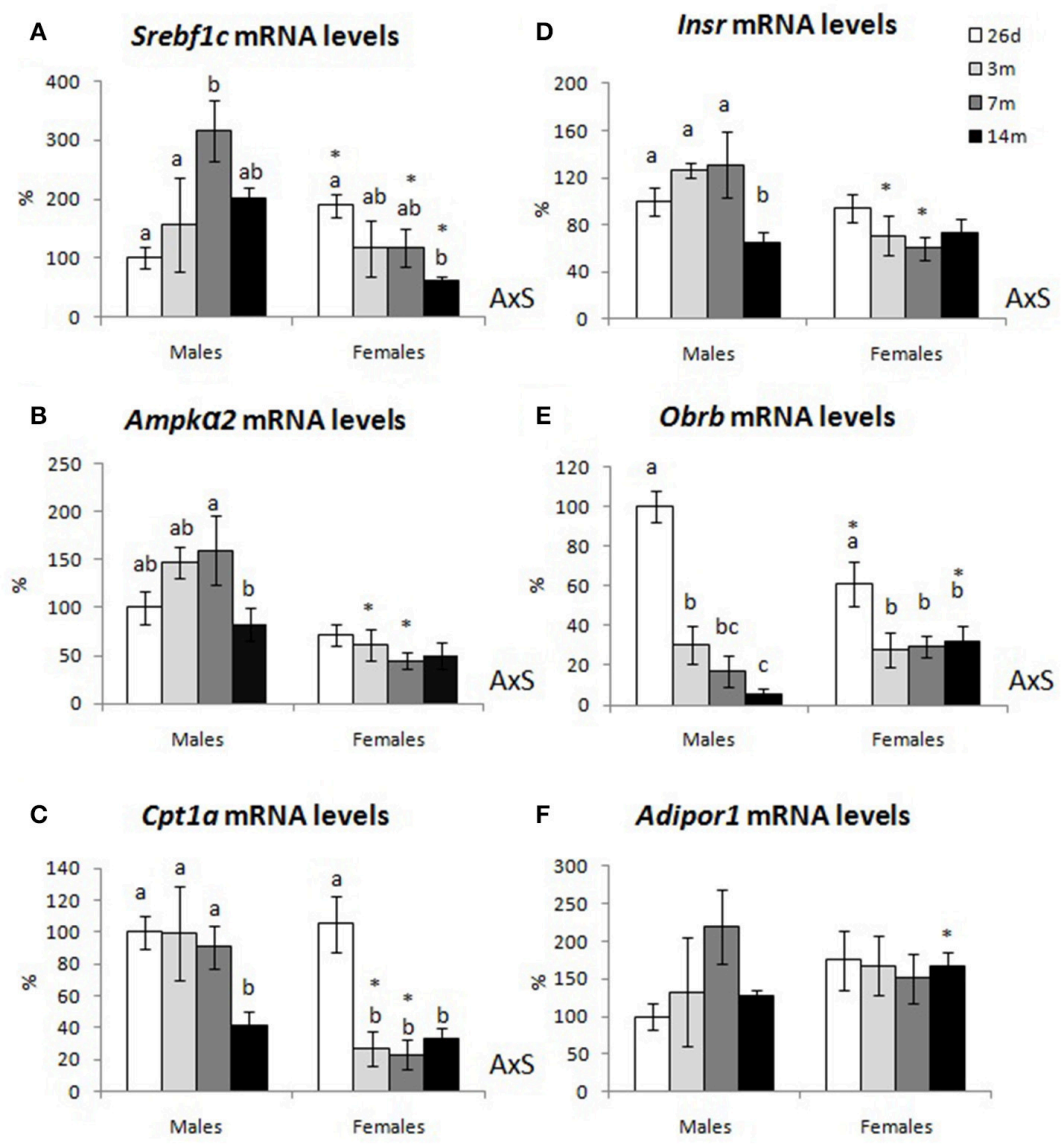
Gene expression levels in liver of male and female animals at 26 days, 3, 7, and 14 months. **(A)** sterol regulatory element-binding protein 1C (Srebf1c), **(B)** AMP-activated protein kinase alpha 2 (Ampkα2), **(C)** carnitine palmitoyltransferase 1 (Cpt1a), **(D)** insulin receptor (Insr), **(E)** leptin receptor (Obrb), and **(F)** adiponectin receptor 1 (Adipor1) mRNA levels are expressed as a percentage of the mean values of 26-day-old males. Data represent mean ± SEM. Statistics: AxS, interaction between age and sex by two-way ANOVA; a ≠ b ≠ c by LSD *post-hoc* analysis. ^*^different from males at the same age by Student's *t*-test.

Figure 7 shows hepatic mRNA expression levels of selected genes related to lipid metabolism—*Srebf1c, AMP-activated protein kinase alpha 2* (*Ampk*α*2) and carnitine palmitoyltransferase 1 liver isoform (Cpt1a)*—and leptin, insulin, and adiponectin signaling (*Obrb, Insr, Adipor1*, respectively) in male and female animals at the different ages studied. Concerning *Srebf1c* (Figure 7A), mRNA expression levels in males peaked at 7 months of age, showing a tendency to decrease afterwards (although differences did not reach statistical significance). In females, *Srebf1c* transcripts levels decreased gradually with age, and levels at 14 months were significantly lower than those at 26 days of age (interaction between sex and age, *P* < 0.05 by two-way ANOVA). mRNA levels of *Ampk*α*2* (Figure 7B), *Cpt1a* (Figure 7C), and *Insr* (Figure 7D) in males remained unchanged until 7 months old, but decreased thereafter. Females showed no changes with age regarding *Ampk*α*2* and *Insr* transcript levels, whereas *Cpt1a* mRNA expression levels decreased significantly at 3 months and remained unchanged from then on (interaction between sex and age, *P* < 0.05 by two-way ANOVA).

Concerning *Obrb* (Figure 7E), its transcript levels in the liver decreased gradually with age in males (levels at 14 months were almost 100 times lower than those of 26-day-old animals), whereas in females they displayed a significant decrease from 26 days to 3 months of age, but levels remained unchanged afterwards (interaction between sex and age, *P* < 0.05 by two-way ANOVA). No significant differences were observed with age concerning *Adipor1* mRNA levels, in either males or females (Figure 7F).

## Discussion

In the present study, we show sex-differences in age-related metabolic disturbances in rats, along with differences in the capacity for WAT expansion and in adiponectin profile and leptin sensitivity. More specifically, parameters related to fat accumulation, such as adiposity index and relative weights of the iWAT, mWAT, and rWAT depots, as well as total DNA content in the tissue, gradually increased with age in female animals, whereas in males these parameters reached stable values at 7 months of age, with no further increase thereafter. It is precisely in this period of time when male animals started showing metabolic alterations such as insulin resistance, greater triglyceridemia, higher hepatic TG content, and inflammation, while female animals were more protected from these complications.

WAT grows by two known mechanisms, hyperplasia (cell number increase) and hypertrophy (cell size increase). Hypertrophy, compared to hyperplasia, is a more harmful way of fat gain, and has been related to macrophage infiltration (Cinti et al., 2005) and metabolic complications (Arner et al., 2010). In this regard, the increase in adipose tissue size with age in females appears to be more mediated by hyperplasia, as deduced by the gradual increase in total DNA content and the greater expression levels of the proliferation marker PCNA found in the inguinal depot. Unlike females, male animals maintained a stable DNA content in their fat depots from 7 months old. Males clearly showed larger adipocytes compared to females at 14 months, and also displayed more evident signs of macrophage infiltration in the retroperitoneal and inguinal depots. Notably, these sex-associated differences in macrophage infiltration have been previously described in diet-induced obese mice (Medrikova et al., 2012).

These results are in line with the “adipose tissue expandability hypothesis,” which states that a failure in the capacity for WAT expansion, rather than obesity *per se*, is an important factor that is potentially involved in the origin of metabolic syndrome complications (Virtue and Vidal-Puig, 2010). According to this hypothesis, the limited capacity of male animals to expand their fat depots may be associated with the greater prevalence of metabolic complications occurring in these animals with aging. Similar findings have been described in animals exposed to an obesogenic environment; the greater capacity of females to respond to a high-fat diet by storing excess fat in WAT has been proposed to account for their protection against hepatic fat deposition and their better insulin sensitivity compared with males (Priego et al., 2008; Medrikova et al., 2012). Studies in other models also support the importance of adipose tissue expandability in the metabolic health of the organism. On the one hand, overexpression of glucose transporter GLUT4 (Shepherd et al., 1993) or mitochondrial protein mitoNEET (Kusminski et al., 2012) in WAT, with the consequent increase in fat mass, has been shown to enhance insulin sensitivity. In turn, increased adipose tissue expandability also resulted in an improved metabolic profile under a high-fat diet (Abreu-Vieira et al., 2015). On the other hand, blockage of adipose tissue expandability is associated with severe metabolic alterations (Medina-Gomez et al., 2007). In this regard, we found that male rats displayed an increase in insulin resistance markers (HOMA-IR index and leptin/adiponectin ratio) and of liver TG content with aging, along with an important drop in mRNA levels of insulin and leptin receptors in liver and WAT. Furthermore, leptin resistance at the adipocyte level due to local reduction of the leptin receptor has been related to metabolic disorders associated with obesity (Huan et al., 2003). Thus, adipocyte-reduction in leptin signaling occurring in aged males may account, at least in part, for the altered insulin signaling and dyslipidemia that these animals experience. In turn, the loss of leptin signaling in liver has been associated with hepatic steatosis (Fishman et al., 2007; Stucchi et al., 2011) and higher TG incorporation into VLDL particles (Huynh et al., 2013). In our study, only males showed an increase in hepatic TG content with aging, which was inversely correlated with transcript levels of *Obrb* in this tissue (*r* = –0.574, *p* < 0.001; Pearson's correlation).

Therefore, mechanisms that control WAT expansion may be of importance regarding the development of lipotoxicity in non-adipose tissues and the appearance of alterations related to the metabolic syndrome. In this regard, *Mest* and *leptin* expression have been used independently as markers of WAT expansion and adipocyte size. On the one hand, MEST protein has been proposed to have a unique role in modulating adipose tissue expansion (Nikonova et al., 2008). Its expression levels in WAT show the strongest association to adipose tissue expansion in comparison to other genes, including *leptin* (Kozak et al., 2010), and, in addition, upregulation of *Mest* after high-fat diet exposure in rodents has been shown to be very rapid and highly reproducible, confirming its role as an early marker of WAT expansion (Voigt et al., 2013). On the other hand, *leptin* mRNA expression in WAT, as well as serum leptin levels, is known to increase with age and to be mainly associated with an increase in adiposity (Oliver et al., 2001) and adipocyte size (Guo et al., 2004). Nevertheless, it has been described that the overload of lipids of a fat depot is accompanied by the attainment of maximal *leptin* mRNA expression levels (Oliver et al., 2001). In the present study, we found sex differences in the expression levels and ontogenic pattern of expression of *Mest* and *leptin* genes in WAT. Specifically, while male animals exhibited a decrease in *Mest* expression levels in the rWAT with age, expression levels in female rats were higher than in males from 7 months onwards and remained stable with age. Regarding leptin, female animals showed a continuous increase in their expression levels with age in the rWAT depot revealing that, in the conditions studied, this tissue has not yet become overloaded. On the other hand, in males, *leptin* expression levels remained constant at the age at which the relative weight of both inguinal and retroperitoneal depots reached the maximum value. From these results, it could be assumed that adipose depots in female animals would still maintain their capacity to increase *leptin* expression in response to an additional accumulation of lipids, whereas this capacity would be exceeded in males. Therefore, although *Mest* and *leptin* expression levels are related to WAT expansion when considered individually, sex differences in the capacity to expand adipose tissue with age would be illustrated in a most precise manner by differences in the *Mest/leptin* mRNA ratio, with high values being indicative of greater adipose storage capacity. In males, this ratio decreased dramatically with age in both adipose depots studied, whereas females peaked at 3 months and, thereafter, maintained values significantly higher than those of males.

The underlying mechanisms whereby females may have greater WAT expandability and exhibit lower propensity than males to age-related alterations are not known, beyond considering the plausible implication of sexual hormones. Estrogen removal in animals or menopause in women is associated with some metabolic disturbances such as hepatic TG accumulation or an increase in HOMA-IR index (Paquette et al., 2007; Völzke et al., 2007; Cote et al., 2012). Aside from sexual hormones, the involvement of melatonin should be considered in the sex-associated differences observed with age. Melatonin levels in humans gradually decline throughout life and this has been suggested to contribute to the incidence or severity of some age-related diseases (Karasek, 2004). Notably, it has been described that females exhibit greater levels of melatonin and a higher amplitude rhythm of secretion in comparison to males (Gunn et al., 2016). Moreover, adiponectin has been proposed to contribute to sex differences in insulin sensitivity (Medrikova et al., 2012), and some of its beneficial metabolic effects have been related to its involvement in WAT expandability (Kim et al., 2007; Asterholm and Scherer, 2010; Medrikova et al., 2012). In this sense, besides the occurrence of higher adiponectin levels in adult female rats compared with males, as anticipated (Medrikova et al., 2012), females maintained adiponectin levels from 7 to 14 months old, while levels in males underwent an important drop during this period of time. Concomitantly to this decrease, some of the detrimental effects of aging (greater insulin resistance, increased circulating TG and their content in liver) became apparent in males but not in females.

Due to its economic costs, only a few *in vivo* studies have been performed to determine the effects of adiponectin. Notably, chronic adiponectin administration in high-fat diet fed rats has been described to improve pathways of insulin signaling and lipid storage in adipose tissue, contributing to a better metabolic profile (Matafome et al., 2014). These experimental data also support the relevance of the maintenance of high levels of adiponectin for the prevention of age-related detrimental effects. Adiponectin action in preventing the development of fatty liver disease may also involve its direct effect in this tissue by suppressing the expression of SREBF1c, which upregulates the main enzymes involved in fatty acid synthesis (Awazawa et al., 2009). This effect seems to be mediated, in part, by the activation of AMPK, through its action on ADIPOR1 (Awazawa et al., 2009). In this regard, we found that 7-month-old male animals displayed increased transcript levels of *Srebf1c* compared with levels at younger ages, while expression levels of this gene steadily decreased with age in females. In addition, the expression levels of genes related to fatty acid oxidation (*Ampk*α*2* and *Cpt1a*) were also decreased in 14-month-old males. These results suggest that adiponectin action in liver may be impaired with age in males, and this fact may contribute to an increase in fatty acid synthesis and a decrease in their oxidation rate, in line with the hepatic accumulation of lipids. Nevertheless, although protein levels are expected to follow gene expression patterns, the regulation at the protein level/activity cannot be excluded. All in all, the present results point out that both increased plasma levels of leptin with aging (indicative of leptin resistance) and decreased levels of adiponectin (due to overloading of adipose tissue and related to lower adipose tissue expandability) may contribute to the higher prevalence of metabolic disturbances in aged males compared with females.

Besides differences in expandability, other processes such as the effect of browning may also be considered in the sex-associated differences found with age. In this regard, it has been described that female animals are more responsive to the recruitment of brown adipocytes in comparison to males (Kim et al., 2016). Brown adipocytes have the capacity to dissipate energy as heat, and the recruitment and activation of this type of adipocytes has been proposed as a new tool against obesity-related metabolic diseases (Lee et al., 2014). This browning process could be of particular importance in the subcutaneous adipose tissue, since this has been described to be the most reactive depot to acquiring brown adipose tissue characteristics when exposed to cold (Rosell et al., 2014), and in the present study animals are housed at standard room temperature conditions (22°C), which is not thermo-neutrality. Thus, the protection from age-related metabolic alterations observed in females may be, to some extent, the result of more active browning processes in these animals, particularly in the iWAT, although this possibility was not analyzed in the present study and needs further investigation.

In conclusion, female rats are more protected from age-related metabolic alterations, such as insulin resistance, hypertriglyceridemia, hepatic lipid deposition, and adipose tissue inflammation, compared with males. It is suggested that this protection may be associated with a greater capacity for WAT expansion—reflected in a higher *Mest*/*leptin* mRNA ratio—and related to their increased ability to sustain elevated adiponectin levels and to preserve leptin sensitivity with aging, hence maintaining a lower circulating leptin/adiponectin ratio.

## Ethics statement

The animal protocol followed in this study was reviewed and approved by the Bioethical Committee of the University of the Balearic Islands (Resolution Number 8453. June 2010), following its guidelines for the use and care of laboratory animals. All efforts were made to minimize suffering.

## Author contributions

FG, TP, and NS carried out the experiments. FG, TP, CP, and AP designed the experiments, evaluated results, and wrote the paper.

### Conflict of interest statement

The authors declare that the research was conducted in the absence of any commercial or financial relationships that could be construed as a potential conflict of interest.
